# Nuances of COVID-19 and Psychosocial Work Environment on Nurses’ Wellbeing: The Mediating Role of Stress and Eustress in Lieu to JD-R Theory

**DOI:** 10.3389/fpsyg.2020.570236

**Published:** 2020-10-29

**Authors:** Tang Meirun, Sobia Bano, Muhammad Umair Javaid, Muhammad Zulqarnain Arshad, Muhammad Umair Shah, Umair Rehman, Zar Ayesha Parvez, Muhammad Ilyas

**Affiliations:** ^1^School of Management, Guizhou University, Guiyang, China; ^2^Department of Management Sciences, GIFT Business School, GIFT University, Gujranwala, Pakistan; ^3^Department of Management Sciences, Lahore Garrison University, Lahore, Pakistan; ^4^Department of Management Sciences, Faculty of Engineering, University of Waterloo, Waterloo, ON, Canada; ^5^Department of User Experience Design, Faculty of Liberal Arts, Wilfrid Laurier University, Branford, ON, Canada; ^6^Department of Management Sciences, Lahore Garrison University, Lahore, Pakistan; ^7^Department of Management & MIS, University of Hail, Thuwal, Saudi Arabia

**Keywords:** psychosocial work environment, COVID-19, JD-R, nurses, developing country

## Abstract

**Background:**

The global spread of COVID-19 makes Pakistan as vulnerable as any other developing country and the risk posed by the weak health system increases the fears in people’s minds. The government is strategically expanding the scope of community ownership and increasing understanding in the population through risk communication and engagement; still, the situation remains very austere and is even affecting the psychological health of caregivers. We, therefore, sought to determine the impact of psychosocial job demands and resources associated with the psychological health of nurses in a time lag duration of 3 months, i.e., since the start of the pandemic, from January to March 2020. We hypothesized the significant mediating roles of stress and eustress in a direct relationship with psychosocial work environment job demands, job resources, and nurses’ wellbeing.

**Methods:**

In this cross-sectional self-administrated study, we distributed the survey in two parts by using a time-lag strategy to collect data at the start of pandemic (Time 1) and then again 3 months later (Time 2). Data on 53 items was collected from 208 female nurses who had participated in both the time phases and met the eligibility protocols of the study (such as being certified female nurses who have a registration number (RN) through the Pakistan Nursing Council (PNC), having 4 years of a generic nursing degree, and 2 years of nursing experience).

**Findings:**

We have achieved three stages through our analytic study on the nurses’ samples to determine the predictive abilities for the quality of the psychosocial work environment model. The coefficient of determination is *R2*, while the effect size is *f*2. We found 29.0% variance, 0.05 and 0.03 effect size, and 0.153 predictive abilities on stress as explained by job demands, and 53.4% variance, 0.19 and 0.39 effect size, and 0.275 predictive abilities on eustress as explained by job resources. And finally, there was 71.2% variance, 0.00, 0.02, 0.02, 0.03, 0.42, and 0.07 effect sizes, and 0.545 predictive abilities on our third endogenous construct, wellbeing, which is explained by both the psychosocial job demands and job resource variables. From partial to full mediation, stress and eustress significantly impact the psychosocial work environment of nurses.

## Introduction

Unfortunately, the concept of a psychosocial work environment is ill-addressed in many developing countries in Asia and is extremely understudied in Pakistan. The situation is so dire that even well-equipped organizations are less interested in addressing the psychosocial risks an employee faces in the workplace. Either these organizations are taking it for granted, or they are seriously unaware of it. The concept of balancing job demands and job resources to create harmony for the employee is almost non-existent in organizations that deal with the public, and of course hospitals are no exception, where health care providers such as doctors and nurses are considered to be the heart and soul of a healthcare system. Every country needs competent, motivated, well-distributed, and supported health staff, such as nurses, who are indeed the unsung heroes and backbone of the primary healthcare system (WHO, 2020). Nurses are considered as the most significant human resource in healthcare organizations. Their 24/7 presence has entirely transformed how healthcare is delivered to patients. Yet, for all its importance, nursing remains underappreciated. Perhaps the most significant barrier that continues to stifle the profession concerns gender and stereotypes (The Lancet, 2019). Among several factors that negatively affects the optimal functioning of nurses, workplace bullying (WPB) is widespread and relatively subtle, but critical, as it increases poor health, stress, and attrition (Finchilescu et al., 2019; The Lancet, 2019). It is commonly considered that bullying starts due to a poor psychosocial work environment (Agervold and Mikkelsen, 2004).

Before the pandemic, hospital caregivers provided routine services to patients. However, healthcare systems have been rocked by the sudden emergence of Coronavirus disease (COVID-19), which as of August has affected more than 200 countries, areas, or territories, with near to 20 million confirmed cases, 720441 deaths, and countless more human beings who have been effected with deteriorating physiological and psychological health and wellbeing issues (WHO, 2020). This global spread makes Pakistan as vulnerable as any other developing country, and the risk posed by a weak health system increases the fears in people’s minds. By August, the country had reported 282645 confirmed COVID-19 cases, out of which there were 6052 confirmed deaths. Though the National Action Plan for COVID-19 Pakistan shows that the government is strategically expanding the scope of community ownership and increasing understanding in the population through risk communication and engagement, the situation remains very austere.

The COVID-19 pandemic and its far-reaching implications continue to unfold globally and, in Pakistan, the focus on the death toll and increasing infection rate has unfortunately eclipsed the massive psychosocial impact on nurses who are experiencing a wide range of thoughts, feelings, and reactions including psychological health issues such as stress, anxiety, anger, and fatigue along with physiological health concerns like increased heart rate, somatic stress, or other uncomfortable sensations. Realistically, significant global public health measures are required to combat the rapid spread of this pandemic with its range of deteriorating health outcomes (Wangping et al., 2020); this is particularly true as this virus has no vaccine yet, meaning the rush of people being infected has led to some nurses being fearful that they may contract it as well, as quarantine alone is not sufficient to control the spread of this pandemic (Sohrabi et al., 2020).

To date, exact data on the psychological health of caregivers during the COVID-19 pandemic is unavailable (Xiang et al., 2020). However, previous research has revealed a profound and broad range of psychological impacts on caregivers (Dewey et al., 2020; Greenberg et al., 2020; Ho et al., 2020; Kang et al., 2020; Lai et al., 2020; Xiang et al., 2020). Among the general public at an individual level, it can precipitate new psychiatric symptoms in people without mental illness, aggravate the condition of those with pre-existing mental illness, and cause distress to the caregivers of affected individuals (Ho et al., 2020). A study conducted on 260 healthcare workers in Italy on different psychosocial work environment factors found that, amongst different constructs, stigma had a high impact on the workers’ outcomes when they interacted with COVID-19 patients (Ramaci et al., 2020). In a cross-sectional study conducted on 1257 healthcare workers from 34 Chinese hospitals, it was found that a considerable proportion of healthcare workers reported experiencing symptoms of depression 50.4%, anxiety 44.6%, insomnia 34%, and distress 71.5% – especially female nurses. This shows nurses are at high risk of developing unfavorable mental health outcomes and may need psychological support and interventions. In a study conducted by Greenberg and colleagues, managing the mental health challenges faced by healthcare workers during the COVID-19 pandemic was discussed and it was recommend that healthcare managers and staff need to proactively look after each other by providing psychological wellbeing. He added that the decisions that caregivers face in this time of uncertainty could significantly enhance psychological pressure, and such decisions normally fall into four categories: allocation of resources, aligning patient needs with the family, caring for severely unwell patients, and balancing the physical and mental health needs of workers (Greenberg et al., 2020). Moreover, earlies studies have identified that COVID-19 is more prevalent among individuals with comorbidities, such as hypertension, diabities, heart disease, breathing issues, and especially aged people (Jiménez-Pavón et al., 2020). The study conducted in Italy found that human age itself plays a critical role in the increased number of pandemic effected cases. The study concluded that 23% of Italy’s elderly population (those aged 65 years or older) have been infected with COVID-19 (Onder et al., 2020). Therefore, at the time of this pandemic, there is a dire need to control psychosocial stressors and increase psychosocial health and wellbeing through behavioral change, psychosocial support, and many different intervention strategies (Ammar et al., 2020a, b; Chtourou et al., 2020).

The JD-R Model (Demerouti et al., 2001) and JD-R theory (Bakker et al., 2014) have gained immense popularity among researches during the twenty-first century. JD-R is classified into two main divisions based on unique properties and characteristics: job demands and job resources. Job demands include physical, psychological, social, or organizational aspects of the job that require psychological cognitive and emotional effort or skills that increase job stress rate (Bakker and Demerouti, 2018). These demands would include a high workload, bullying, demands of any nature, from quantitative to emotional, complex tasks, conflicts, an unfavorable environment, and irregular working hours. Job demands could very well turn into job stressors when meeting those demands needs high effort, and from which the employee fails to recover. So, job demands are the facets of work that decreases employee energy. Job resources include those physical, psychological, and social facets of the job that help in achieving work goals, reducing job demands, and stimulate personal growth, learning, and development of employees (Bakker and Demerouti, 2018). Job resources are the facets of work that help employees to achieve their goals and handle job demands well (Demerouti and Bakker, 2011). These include self-determination, creating an environment for conflict management, increased organizational justice for employees, good performance feedback, and social support. These are motivating job characteristics as these satisfy an employee’s basic psychological needs. Therefore, job demands and resources have independent effects on employee well-being. While job demands may affect physical health and wellbeing in various ways, like increased work pressure, job resources are motivating and contribute positively to employees’ health and wellbeing (Demerouti and Bakker, 2011).

The literature further exhibits that psychological health and wellbeing is a complex concept that is influenced by various psychosocial factors and is often related to quantitative demands, emotional demands, work-family conflicts, job insecurity, individual traits, coping mechanisms, and the resources available at personal and organization level (Rasulzada, 2007; Javaid et al., 2019). Wellbeing comprises of various sources of satisfaction from different areas of life, which includes personal life, like leisure activities and family and social life, satisfaction from job/work-related aspects like salary, promotion, and co-workers, and general health. Within the domain of behavioral sciences, specifically occupational health psychology, a lot of emphasis is given to the wellbeing of employees, both physical and mental (Avey et al., 2010). The dynamics of employee wellbeing play a paramount role in identifying the areas of work which affects quality of life at work (Baptiste, 2008). Research on poor psychosocial work environments is of the utmost importance; for example, it has been demonstrated that a fear of reporting problems can result in poor mental health and wellbeing. A poor psychosocial work environment may have tragic consequences on industry workers as well as patient care (Balducci et al., 2012; Islamoska et al., 2018; Javaid et al., 2018).

The JD-R has been supported in studies within healthcare environments, whereby the main purpose of this study is to identify the dominance of psychosocial job demands and job resources on the wellbeing of nurses with an indirect effect on psychological health factors. In lieu of the JD-R theory, we have hypothesized that psychosocial job demand variables (workplace bullying and emotional demands) positively predict stress and negatively predict wellbeing, i.e., H1, H2, H3, and H4. In addition to that, we hypothesized that psychosocial job resource variables (a climate for conflict management and organizational justice) positively predicts eustress and wellbeing, i.e., H5, H6, H7, and H8. In this study, we also hypothesized on the direct effect of stress and eustress on wellbeing, i.e., H9 and H10. We also hypothesized that the dominance of psychosocial job demands (workplace bullying and emotional demands) and job resources (climate for conflict management and organizational justice) on the wellbeing of the nurses has an indirect effect on psychological health factors, which are stress and eustress, i.e., H11, H12, H13, and H14.

## Methodology

### Participants

This is indeed an unprecedented time for all of us, especially to the current generation, therefore, to evaluate the real impact on psychological health, we have planned to take data from participants within the age bracket of 25–45 years. The study participants were female nurses working in different hospitals in Punjab, Pakistan. As per the analysis by WHO of 104 countries, roughly 70% of the global healthcare force is made up of women. Therefore, the researchers have targeted data from female nurses only. Also, according to the state of the world nursing report 2020 by WHO, in Pakistan, nurses make up 25.7% of the healthcare workforce, with 81% of the nurses being female, with 53% aged less than 35 years, and 38% falling in the age bracket of 35–54 years. To the latest year, there are a total of 103,777 nursing professionals; 5600 nurses graduated per year by availing a minimum of 4 years duration of nursing degree. The inclusion criteria is based on three reasons. One, the most populated province was selected, which is Punjab, with the highest number of hospitals (388) (PBS, 2018), and also due to pandemic lockdown, it was difficult to travel in a group. Second, only certified female nurses who had a registration number (RN) through Pakistan Nursing Council (PNC) were able to participate and, finally, nurses who were the part of the study sample had to have a minimum of 2 years of experience in the nursing profession, which includes a 1-year internship, after their 4 years of generic nursing degree registered with PNC. All female nurses who had failed to meet the inclusion criteria were excluded from the survey.

### Sampling Design

Nurses are considered to be an intermediary between the doctor and the patient and are generally with patients 24/7. It is difficult for nurses to spare time for any other activity, particularly during nursing hours. The researchers have considered the complexity of their profession when constructing the long study survey, which consists of 53 items, particularly during this global pandemic (which was declared as such on January 30th, 2020). Therefore, a face to face self-administered questionnaire survey technique was used. In order to minimize bias at the time of data collection, we ensured that respondents maintained the social distance of two meters and filled the questionnaire independently in the presence of the research team. It was decided to separate the survey into two parts by using a time-lag strategy, i.e., data were collected in two phases using a 3 months’ time lag (Idris and Dollard, 2014), during January and March 2020. In the first phase (Time 1), the survey was comprised of variables, such as workplace bullying, emotional demands, and stress, with a combined 34 items. The second phase of the survey was conducted after 3 months (Time 2) in which organizational justice, climate for conflict management, and eustress were the variables, with 19 items in total. It All participants were ensured that the information provided by them in the current survey would remain confidential. The hospitals were selected based on the convenience of the researcher, and the snowball sampling technique was used to collect data from nurses. This sampling technique is also called referral sampling. In this technique, the existing study subjects recruit future subjects from among their acquaintances. Thus, the sample group is said to grow like a rolling snowball. A total of 290 self-administrated surveys were distributed in the first phase (Time 1), out of which 247 were completed. In the second phase (Time 2), the same 247 nurses were contacted for a survey, out of which 208 responded, and the remaining 39 nurses have excused the researchers because of their professional commitments. Therefore, finally, 208 were considered for data analysis. The valid response rate of the survey was 71.7% (208/290^×^100). We examined the potential difference between the two groups, i.e., the first group (*n* = 208) responded in both surveys, the second group (*n* = 39) were those who did not respond in the second survey. The results (*p* > 0.05) indicated that the two groups did not differ regarding their mean scores on outcomes variables, nor did they differ regarding their demographic characteristics. Some of the demographic characteristics of the nurses are as follows.

According to the data, the highest number of respondents, 92 (44.2%), are between 31 and 40 years of age. This is followed by the fact that 85 of them (41.0%) are within the 21–30 age bracket. While 17 of the respondents (8.2%) fall between the 41–50 years of age bracket, only 14 (6.8%) are above 50 years of age. In addition, general nursing among the respondents constitute 38.4%, representing 84 respondents, while BA nursing constitutes 8.5% (18 respondents), and 13.9% (29 respondents) have BS nursing. The remaining forms of nursing are comprised of DCS 9 (4.3%), DHMS 32 (15.4%), DLHV 35 (17%), and intermediate 5 (2.5%).

Furthermore, a total of 79 (38.0%) are staff nurses in their respected organizations, while 47 (22.8%) of them are general nurses. In addition, 76 (36.4%) are the in-charge nurse in the hospital—finally, six (2.8%) nurses are working as a senior nurse. In addition to that, the nurses work in seven different departments. OTA has the majority contribution 64 (30.9%), OPD 63 (30.3%), Gynae 50 (24.0%), Nursery 16 (7.1%), Emergency 8 (4.2%), Administration 5 (2.5%), and Medical 2(1%) in the hospitals.

### Survey Translation

The current study was conducted in Urdu, the national language of Pakistan; therefore, all the study variables were translated into Urdu from English using the back-translation technique. The forward-then-back translation procedure was completed as per the recommendations of (Isha et al., 2020). Further, to ensure that the contents of each item were cross-linguistically comparable and generated the same meaning, we have used both translated languages in a single questionnaire.

### Survey Measures

In the workplace, bullying refers to harassing, offending, socially excluding someone, or negatively affecting someone’s work tasks. The scale of workplace bullying was adapted from Einarsen et al. (2009), and is comprised of three dimensions. The first dimension is of *work-related bullying (WRB)* has 7 items, such as ‘does someone withhold information which affects your performance in work?’ translated into Urdu as ہے؟ س ک تی ہ و م تاث ر ک اک ردگ ی م یں ک ام پ کیآ سے ہ ون ے مع لوم ک چھ ک ودو سرے ک سی م یں ب ارے ک ے پآ ک یا Item WRB7 was deleted because of low factor loading i.e., <0.7. The second dimension is *person-related bullying (PRB)* which has 12 items, such as ‘Have you been humiliated or ridiculed in connection with your work?’ translated into Urdu as ہے؟ پ ڑا ک رن اسام نا ک ا حال ت خ یز مضح کہ ی ا م یزآ ذل ت م ت ع لق سے ک ام ک و پآ ک یا. The third dimension is *physically intimidating bullying (PIB)* which has 3 items, such as ‘Have you been shouted at or been the target of spontaneous anger from your colleagues during work?’ translated into Urdu as ہ یں؟ ب نات ے ن شان ہ ک ا غ صہ معمول ی غ یر ی اہ یں چلات ے پ ر پآ سات ھی ک ار ہ م ک ے پآ دوران ک ے ک ام ک یا. The overall construct composite reliability was 0.961. *Emotional demands* refers to dealing with strong feelings such as sorrow, anger, desperation, and frustration at work (Johannessen et al., 2013). The scale was adapted from Duan-Porter et al. (2018) which consists of a total of 7 items, such as ‘Are you required to treat everyone equally, even if you do not feel like it?’ translated into Urdu as ہ وں ک رت ے ن ہب ھی پ س ند ا سے پآ ےہاچ، ہے جات ا ک یا مطال بہ ک ا ک رن ے س لوکم ساوی ان ہ سے ای ک ہ ر سے پآ ک یا. The overall construct composite reliability was 0.865. *Organizational justice* refers to rules and norms that govern the company in terms of distribution of resources and benefits (distributive justice), processes and conditioning related to these resources (procedural justice), interpersonal relationships (interpersonal justice), and communication and keeping employees informed (informational justice). The scale of organizational justice was adapted from O’Sullivan (2011), which comprised 4 items, such as ‘Are conflicts resolved in a fair way?’ translated into Urdu as ل وگ وں دو سرے م یںہے؟ جات ا ک یا حل م یں ان داز ان ہم ن صفک و ت نازعات ک یا. The overall construct composite reliability was 0.851. *Climate for conflict management* refers to the employees’ belief that interpersonal conflicts are generally managed well and fairly in their organization, and that general procedures for the distribution of benefits and burdens in the organization are fair (Einarsen et al., 2018). The scale of climate for conflict management contains five items, such as ‘I have not been able to stand dealing with other people’ translated into Urdu as ہ وں رہ تی ش کار ک ا دب او ذہ نی عموماَ م یں م یں خ یال م یرےہ وں ن ہ یں ت یار ل یےک ےن م ڻ نے سے. The overall construct composite reliability was 0869. *Stress* refers to a change in an individual’s natural equilibrium state (severe pressure of pain, or sorrow and anguish, or breathlessness). The scale of stress was adapted from O’Sullivan (2011) which consists of 5 items, such as ‘I generally view myself as being stressed’ translated into Urdu as ہ یں ل ی تے ن مٹ سے طری قے اک ثرموث ر سےت غ یر ک ے دب او وال ے آنےپ یش م یں زن دگ ی اپ نی آپ. The overall construct composite reliability was 0.896. *Eustress* refers to the adaptation response toward a stressor, which is perceived as positive by the individual. The scale of eustress was adapted from O’Sullivan (2011) which consists of 10 items, such as ‘How often do you effectively cope with stressful changes that occur in your life?’ translated into Urdu as مجموعی طور پر ہر چیز. The overall construct composite reliability was 0.926. *Wellbeing* refers to good and satisfactory conditions of existence or a state characterized by health, happiness, and prosperity and is measured by 5 items, such as ‘how pleased are you with your job as a whole, everything taken into consideration?’ translated into Urdu as ملازمت سے ک ت نے خوش ہ یں’. پ اپ نیکو ذہن میں لاتے ہویٴے آ. The overall construct composite reliability was 0.863.

## Statistical Analysis and Results

### Measurement Model

The hierarchical component modeling technique used as one of the constructs is higher-order, i.e., workplace bullying, and the model is Reflective-Reflective. In the measurement model for reliability and validity, we have run confirmatory factor analysis (Arshad and Arshad, 2019). First, we assessed the individual item loading of the constructs. All the loadings are more than 0.6, which is the threshold value, except for one item of workplace bullying, i.e., WRB7, which was below 0.4. This item was then deleted from the model. Thus, the indicator reliability of each construct was established.

After that, Cronbach Alpha (CA) and Composite reliability (CR) values were assessed. CA values of all the constructs ranged from 0.767 to 0.958 and CR values ranged from 0.865 to 0.961, as shown in Table 1, which are in an acceptable range, i.e., greater than 0.706 (Hair et al., 2016). This indicates that all the constructs in this study have a high level of internal consistency reliability. Next we assessed the Convergent validity of the measurement model. Convergent validity is the degree to which individual indicators correlated to other indicators of a similar construct (Hair et al., 2016). Average Variance Extracted (AVE) is defined as the grand mean value of the squared loadings of the indicators associated with the construct. It reflects the extent to which a construct explains the variance of its indicators. Generally, an AVE value of more than 0.50 indicates that more than 50% of an indicator’s variance can be explained by the construct (Fornell and Larcker, 1981; Bagozzi and Yi, 1988; Hair et al., 2016). Based on Table 1, this study found that the AVE values in this study ranged from 0.518 to 0.795, indicating that the convergent validity of each construct was established.

**TABLE 1:** Summary statistics of the measurement analysis.

	CA	Items Loading	CR	AVE	VIF
CCM	0.799	0.756 – 0.819	0.869	0.624	2.351
ED	0.833	0.662 – 0.805	0.865	0.518	2.868
EST	0.911	0.700 – 0.876	0.926	0.557	2.469
ORJ	0.767	0.705 – 0.816	0.851	0.589	2.519
STR	0.855	0.786 – 0.807	0.896	0.632	2.637
WEB	0.863	0.767 – 0.823	0.901	0.646	1.879
WPB	0.958	0.734 – 0.852	0.961	0.532	2.235
PIB	0.871	0.789 – 0.899	0.921	0.795	2.587
PRB	0.934	0.744 – 0.843	0.943	0.559	2.423
WRB	0.887	0.732 – 0.899	0.914	0.639	2.789

*CCM, Climate for Conflict Management; ED, Emotional Demands; EST, Eustress; ORJ, Organizational Justice; STR, Stress; WEB, Wellbeing; WPB, Workplace Bullying; PIB, Physically Intimidating Bullying; PRB, Person Related Bullying; WRB, Work-related Bullying.*

Discriminant validity refers to the degree to which a construct distinguishes from other constructs by statistical standards (Hair et al., 2016). Fornell and Larcker criterion were used to evaluate discriminant validity, and Heterotrait-Monotrait ratio (HTMT) are examined in this study as shown in Tables 2, 3. In Fornell and Larcker criterion the diagonal value of the construct should be higher than any value of any other construct. For Heterotrait-Monotrait ratio evaluation, any value of constructs should be less than 0.9, which means every construct is distinct from other constructs. The approach, which has been used to detect the multicollinearity, is to check the Variance Inflated Factor (VIF). VIF values less than 5 is acceptable, which shows that no multicollinearity exists (Hair et al., 2016). In this study, results show that no multicollinearity exists in the data set as VIF values were not more than 3, as shown in Table 1. All the criteria for the measurement model have been achieved. This allows us to enter into the next step in which we have assessed the structural model.

**TABLE 2:** Fornell-Larcker criterion.

	CCM	ED	EST	ORJ	STR	WEB	WPB	PIB	PRB	WRB
CCM	0.790									
ED	–0.231	0.719								
EST	0.665	–0.192	0.746							
ORJ	0.493	–0.190	0.591	0.768						
STR	–0.537	0.207	–0.690	–0.497	0.795					
WEB	0.613	–0.279	0.729	0.550	–0.788	0.804				
WPB	–0.164	0.172	–0.200	–0.121	0.256	–0.220	0.729			
PIB	–0.197	0.203	–0.195	–0.136	0.215	–0.202	0.650	0.892		
PRB	–0.140	0.137	–0.189	–0.115	0.237	–0.195	0.370	0.761	0.748	
WRB	–0.150	0.179	–0.174	–0.093	0.255	–0.226	0.607	0.722	0.603	0.799

**TABLE 3:** HTMT criterion.

	CCM	ED	EST	ORJ	STR	WEB	WPB	PIB	PRB	WRB
CCM	–									
ED	0.249									
EST	0.778	0.186								
ORJ	0.623	0.214	0.705							
STR	0.647	0.208	0.781	0.612						
WEB	0.736	0.279	0.820	0.673	0.916					
WPB	0.189	0.189	0.213	0.148	0.283	0.240				
PIB	0.237	0.247	0.217	0.169	0.250	0.232	0.824			
PRB	0.167	0.156	0.205	0.146	0.266	0.216	0.028	0.845		
WRB	0.181	0.195	0.195	0.117	0.294	0.256	0.782	0.821	0.883	–

### Structural Model

The Smart-PLS provides an inner-model evaluation of the path coefficients, effect size (*f*2), and coefficient of determination (*R*^2^ value) of the model. In Smart-PLS, the bootstrapping procedure enables researchers to compute empirical Beta-values, *T*-values, and *P*-values for all structural model assessments. In this study, the researcher used PLS bootstrapping 500 samples size with one-tailed tests of significance level 5% to test the hypotheses.

**FIGURE 1:**
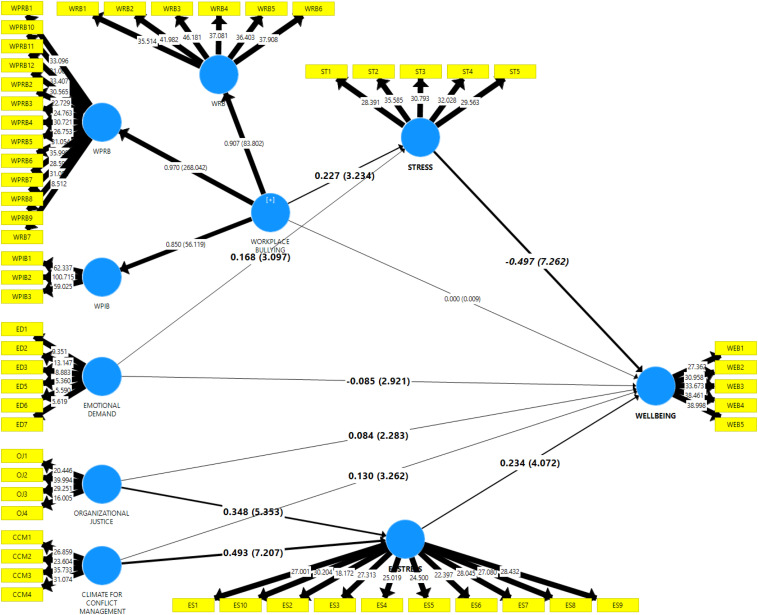
Structural model.

In Table 4 and Figure 1, this study found that workplace bullying positively predicts stress (β = −0.277, *t* = 3.618, *p* < 0.01), supporting H1; workplace bullying did not negatively predict wellbeing (β = −0.000, *t* = 0.009, *p* > 0.05), not supporting H2; emotional demand positively predicts stress (β = 0.168, *t* = 3.344, *p* < 0.01) supporting H3; emotional demand negatively predicts wellbeing (β = −0085, *t* = 3.166, *p* < 0.05), supporting H4. Organizational justice positively predicts eustress (β = −0.348, *t* = 5.059, *p* < 0.01), supporting H5; organizational justice positively predicts wellbeing (β = −0.084, *t* = 2.280, *p* < 0.05), supporting H6; climate for conflict management positively predicts eustress (β = 0.493, *t* = 6.865, *p* < 0.01), supporting H7; climate for conflict management positively predicts wellbeing (β = 0.130, *t* = 3.231, *p* < 0.05), supporting H8; stress negatively predicts wellbeing (β = −0.497, *t* = 6.848, *p* < 0.01), not supporting H9; eustress positively predicts wellbeing (β = 0.234, *t* = 3.744, *p* < 0.01), supporting H10.

**TABLE 4:** Direct hypothesis testing.

Sr. no.	Hypothesis	Beta	Standard error	*T*-value	*P*-value
1	Workplace Bullying - > Stress	**0.227**	**0.063**	**3.618**	**0.000**
2	Workplace Bullying - > Wellbeing	0.000	0.027	0.009	0.496
3	Emotional Demand - > Stress	**0.168**	**0.050**	**3.344**	**0.000**
4	Emotional Demand - > Wellbeing	**−0.085**	**0.027**	**3.166**	**0.001**
5	Organizational Justice - > Eustress	**0.348**	**0.069**	**5.059**	**0.000**
6	Organizational Justice - > Wellbeing	**0.084**	**0.037**	**2.280**	**0.012**
7	Climate for Conflict Management - > Eustress	**0.493**	**0.072**	**6.865**	**0.000**
8	Climate for Conflict Management - > Wellbeing	**0.130**	**0.040**	**3.231**	**0.001**
9	Stress - > Wellbeing	**−0.497**	**0.073**	**6.848**	**0.000**
10	Eustress - > Wellbeing	**0.234**	**0.062**	**3.744**	**0.000**

The coefficient of determination (*R*^2^-value) is the most common measure applied to evaluate the models’ predictive power. The value of *R*^2^ represents the exogenous latent variables combined effects on the endogenous latent variable (Hair et al., 2016). *R*^2^-values of 0.75, 0.50, or 0.25 for endogenous latent variables are described as a substantial, moderate, or weak coefficients of determination, respectively. In the current study, there are three endogenous variables, i.e., Stress, Eustress, and Wellbeing, so the value of *R*^2^ is 0.290, 0.534, and 0.712, respectively. It means that 29.0% of the variance in stress is explained by workplace bullying and emotional demand. Meanwhile, 53.4% of the variance in eustress is explained by organizational justice and climate for conflict management. In addition to that, 71.2% of the variance in wellbeing is explained by workplace bullying, emotional demand, organizational justice, climate for conflict management, stress, and eustress.

Furthermore, this study also assesses the *f*^2^ effect size value, which is explained as the exogenous variable contribution to *R*^2^ values of the endogenous variable (Chin, 1998). The *f*^2^ effect size value of 0.02, 0.15, and 0.35 represent small, medium, and large effects of the exogenous latent variable, respectively. The endogenous variable (stress) is explained by workplace bullying and emotional demands with effect size *f*^2^ of 0.05 and 0.03, respectively, thus indicating a small to medium effect size. In addition, the endogenous variable (eustress) is explained by organizational justice and climate for conflict management with effect size *f*^2^ of 0.19 and 0.39, respectively, thus indicating a medium and large effect size. And the endogenous variable (wellbeing) is explained by workplace bullying, emotional demands, organizational justice, climate for conflict management, stress, and eustress effect size *f*^2^ of 0.00, 0.02, 0.02, 0.03, 0.42, and 0.07 respectively, thus indicating that workplace bullying has no effect. But emotional demands, organizational justice, climate for conflict management, and eustress has a small effect size, and stress has a high effect. Following the steps of the mediator analysis procedure suggested by Hair et al. (2016), as well as running bootstrapping of 500 samples with two-tailed tests of 5% significance level in the PLS-SEM model, this study further determines the mediating effects and its types. Table 5 details the indirect effects of the mediation model.

**TABLE 5:** Indirect hypothesis testing.

Sr. no.	Hypothesis	Beta	Standard error	*T*-value	*P*-value	Result
11	Workplace Bullying - > Stress - > Wellbeing	**0.115**	**0.033**	**3.472**	**0.000**	Partial Mediation
12	Emotional Demands - > Stress - > Wellbeing	**0.081**	**0.030**	**2.756**	**0.003**	Partial Mediation
13	Organizational Justice - > Eustress - > Wellbeing	**−0.083**	**0.026**	**3.194**	**0.001**	Partial Mediation
14	Climate for Conflict Management - > Eustress - > Wellbeing	**−0.113**	**0.035**	**3.188**	**0.001**	Full Mediation

According to Table 5, this study found that the indirect effect of, (workplace bullying - > stress - > wellbeing), (emotional demand - > stress - > wellbeing), (organizational justice - > eustress - > wellbeing), and (climate for conflict management - > eustress - > wellbeing) are significant with (β = −0.115, *t* = 3.472, *p* < 0.01), (β = 0.081, *t* = 2.756, *p* < 0.01), (β = −0.083, *t* = 3.194, *p* < 0.01), and (β = −0.113, *t* = 3.188, *p* < 0.01) respectively. Therefore H11, H12, H13, and H14 are supported. H11, H12, and H13 mediation are partially supported because their direct relationships are also significant. H14 is fully mediated because the direct relationship of workplace bullying and wellbeing is not significant, and it will become significant with the indirect path.

## Discussion, Recommendations and Conclusion

As per the reports of the World Health Organization, the World Health Assembly has designated the current year as the international year for nurses. The importance of a healthy nurse can be seen, as the world needs 9 million more nurses and midwives if it is to achieve universal health coverage by 2030. For Pakistan, with around 5600 nursing graduates per year, this would predict a shortage range of 500,000–600,000 nurses by the year 2030 as compared to a benchmark density. This means psychologically stable nurses in a psychosocial work environment are indeed significantly important to meet the target numbers.

To breakdown the overall objective of this study, the purpose of this research in lieu of JD-R theory was to study how psychosocial job demand factors (workplace bullying and emotional demand) affect the psychological health factor (stress) and wellbeing of female nurses. The psychosocial job demands demonstrated the defacement of psychological health that further affects the mental and physical resources of workers, which contributes to the depletion of energy for workers in the psychosocial workplace environment. As shown by our study results, there is a significant impact of psychosocial, emotional demands, and WPB on the wellbeing of nurses through partial mediation of stress. Besides psychosocial job demands, this research in lieu of JD-R theory also investigated psychosocial job resource factors (organizational justice and climate for conflict management) that affect the psychological health factor (eustress) and physiological health factor (wellbeing) of female nurses. We have also found a significant impact from psychosocial job resources – organizational justice and climate of conflict management - on the wellbeing of nurses, respectively, through partial and full mediation of eustress. However, there is no direct relationship of WPB with wellbeing and it is only significant if stress mediates the two. The possible reasons for insignificant WPB on wellbeing could be due to the small nursing sample size, total tenure of nurses in hospitals, and, in particular, the availability of the opposite gender in those hospitals from where the data was collected. The significant results of psychosocial job resources depict that the system increases moral satisfaction, job commitment, and job satisfaction between nurses, and improves the overall psychosocial work environment. Therefore, we can safely say that nurses should be front and center in terms of attention and protection. An immediate action plan is required to mitigate the negative effects of such risks and those plans must come from the Pakistani government and be implemented by hospitals and followed by caregivers. It is important to deal with the psychological impact of the pandemic upon nurses.

This research was investigated in the hospital of one province; therefore, the results of the data was limited to this location. Many hospitals have a large number of diverse female nurses, so they have more potential participants. Furthermore, the researcher constricted the respondents only to female nurses who met the criteria of the research. This population limited the generalizability of the findings. Future studies should consider these limitations and use different psychosocial work environment variables, especially sleep deprivation, to derive more diverse insights on nurses’ wellbeing. Keeping in view the limitations and results of this study, the authors have recommended a few interventions to maintain a healthy psychosocial work environment, which are addressed below.

### Self-Neglect vs. Self-Management

One of the reasons for nurses being so psychologically stressed is the concept of ‘self-neglect’ in their lives, particularly with the type of environment they are working in and the routine they usually follow. Their 24/7 presence has entirely transformed how healthcare is delivered to patients, and it is justified with the empathetic role they play, but it also appears that on one side some experts and authors regard self-neglect as meaning that an individual is deficient in self-care, whereas others put more emphasis on difficulties in functioning within an environment. For nurses, it could be the hallmark of mental illness and signify a decline into serious health and wellbeing issues. The situation for nurses suffering self-neglect gets worse when significant ethical, personal, and professional challenges arise in which they encounter people such as co-workers and patients in the psychosocial work environment who experiences the same set of mental disabilities. Therefore, establishing an alternating shift system will be a good option to allow nurses periods of rest and ensure that there will be a rotation of workers in high-pressure roles.

### Making a Sound Decision

Healthcare judgment represents the nurse’s likelihood to make sound decisions that are based on the disciplined functioning of mind and emotions. Each nurse is endowed with a unique potential to develop self-sustaining and self-management resources. Self-awareness and self-acceptance are essential to personal integrity and self-worth, and that too can only be achieved if nurses have a psychosocial work environment that contains the climate of conflict management and organizational justice. In providing care, a nurse exercises sound judgment through deliberative, practiced, and educated recognition of sentinel symptoms. Here, the patient’s perception of the situation is an important consideration for the nurse when providing competent care. The more positivity the patient offers the nurse, the more emotional attachment the nurse will feel. Therefore, providing more training in terms of managing psychological problems and post-traumatic stress disorder and/or developing online platforms to provide medical advice to patients and the general public to reduce pressure on nurses working in hospitals is advised.

### Human-to-Human Transaction

A caring occasion occurs when people in the psychosocial work environment come together with their unique qualities to make human-to-human transactions. A caring moment consists of actions and choices made by nurses through interactions with patients in particular and people in the hospital in general. The hospital administration can bring justice by reducing non-critical work activities such as routine follow-ups and non-essential administrative tasks. Also, well-defined and easily accessible protocols and expectations for nurses and the provision of adequate information on stress management, identification of burnout, and other support services for nurses to make them realize that the administration is behind them are recommended. Moreover, recent studies have also highlighted that to mitigate the effect of the pandemic on psychological wellbeing, individuals should increase either indoor or outdoor physical activities while maintaining social distance (Ammar et al., 2020a, b; Chtourou et al., 2020).

### Nursing Work and the Modern Hospital

In Pakistan, the concept of the modern hospital is very limited, and very few hospitals, either public or private, meet those criteria. There is a growing recognition that, as healthcare work becomes increasingly concentrated and multifaceted, excellent patient care depends not on individual brilliance but instead on the coordination of activities to ensure that the appropriate configuration of actors – actions, technology, expertise, materials – are in place to support individual needs. This requires organizational work. The delivery and organization of healthcare is challenging. Recent decades have witnessed an explosion of technologies that draw on systems engineering and management science to rationalize service processes and work activity. Nurses have contributed to these developments, emerging as the lead professionals in the implementation of formal tools such as local protocols and integrated care pathways. Yet, healthcare work often defies such progress due to standardization and control. Individual disease processes can be unpredictable, and many patients have co-morbidities and multifarious needs, which are a poor fit with standardized models. Even in care settings where alignment with a formal plan is possible, the care of individuals takes place in organizations responsible for clinical populations. As such, patients are in competition with each other for access to services, facilities, and the time and attention of health professionals. Sometimes this can be managed systematically through formal systems of triage and scheduling, but often not; hospitals are less able to control their inputs than other industries.

### Situational Challenges of Match-Making

In cases of time-pressured situations, such as intense demands or/and constraint of resources in the workplace, nurses need to be extremely stable both psychologically and physiologically to ensure proper match-making of available job demands and resources. While we discussed on call with one of the senior nurses in the hospital at the time of data collection, we have observed that in such a situation, doctors make autonomous decisions about accepting patients without considering the actual availability of space, which further creates stressful situations for nursing staff. Such situations are worse in developing countries such as Pakistan, where nurses throughout the hospital/clinics are confronted with the need to balance their work-family life along with WPB challenges. Therefore, it is very important to promote communication of such challenges through a range of different forums, to provide access to on-call rooms for nurses who are working long shifts, or even by providing computer or phone-based activities for nurses who are unable to attend work due to mandatory isolation and illness. Once the time-pressured situation is over, it is important for management to ensure adequate time is made to reflect on and learn from the stressful situation.

## Study Implications

During the global pandemic, nurses experienced a variety of psychosocial work environment challenges, that warrants more support and attention from policymakers. The intervening psychosocial job resources, such as managing organizational justice and highlighting a climate of conflict management, in conjunction with the recommendations provided by the authors in this study, offers a baseline for decision makers to create such interventions at organizational and national levels where the health and wellbeing of healthcare workers working at any level can positively be achieved during and after the pandamic. Moreover, intervening psychosocial job resources will be helpful for policymakers; this applies particularly to the Government of Pakistan who have strategically developed and launched the National Guidelines for Infection Prevention and Control and inaugurated a new Center for Occupational and Patient Safety (COPS) at the National Institute of Health (NIH). The centre provides strategic direction for health professionals to embrace, create, and implement transformational changes in the neglected domain of occupational health and safety. The study results can also be useful in crafting resourceful psychosocial work environment practices at a preliminary stage, especially for those nurses who tested positive and were quarantined and must return to their duties to attend patients right after their recovery. This is an all-hands-on-deck moment, therefore actions need to be taken for the wellbeing of nurses in particular and healthcare workers in general and hospitals in Pakistan must empower their health professionals to ensure safe care.

## Data Availability Statement

The raw data supporting the conclusions of this article will be made available by the authors, without undue reservation.

## Ethics Statement

Ethical review and approval was not required for the study on human participants in accordance with the local legislation and institutional requirements. Written informed consent from the [patients/participants or patients/participants legal guardian/next of kin] was not required to participate in this study in accordance with the national legislation and the institutional requirements.

## Author Contributions

TM, SB, and MJ wrote the manuscript. MA and ZP gathered the data. MA analyzed the data. MS and UR helped to address the recommended corrections by the reviewers. All the authors have read and approved the final manuscript.

## Conflict of Interest

The authors declare that the research was conducted in the absence of any commercial or financial relationships that could be construed as a potential conflict of interest.
